# Five Cases Illustrating the Action of Intra-Pulmonary Injections

**Published:** 1885-09

**Authors:** R. Shingleton Smith

**Affiliations:** Physician to the Bristol Royal Infirmary


### Clinical Records.

---

### FIVE CASES ILLUSTRATING THE ACTION OF INTRA-PULMONARY INJECTIONS. By R. Shingleton Smith, M.D. (Lond.), B.Sc., F.R.C.P.; Physician to the Bristol Royal Infirmary.

The following five cases are the only ones in which I have carried out the practice of injecting a solution of iodoform into the substance of consolidated or disintegrating lung. The injections have been performed after the manner practised by Pepper, of Pennsylvania, and advocated by Beverley Robinson, of New York, in the *New York Medical Record* for January 10th, 1885. An ordinary hypodermic syringe has been the instrument employed, and the fluid used has been, in all the cases except one, an ethereal solution of iodoform, five minims containing one grain. The quantity injected has been five to ten minims, and the injections have been repeated daily or at longer intervals.

The injections have been given forty-two times in five independent cases. On the first four occasions the iodoform was dissolved in olive oil; but, in order to avoid any little risk of fat embolism which might arise from the injection of oil into a large blood-vessel, the solution in ether was afterwards employed.

I have been unwilling to use any other substance for injection. The bichloride of mercury, although a more powerful germicide, is so irritating when injected subcutaneously, that its use is not likely to be devoid of danger when injected into the lung texture. Treatment by iodoform has already given encouraging results in the hands of Dreschfeld and others. In a series of cases published in the *Transactions of the International Medical Congress*, of 1884, I found that 29 of the 46 cases showed an absolute gain in body weight, amounting in one case to 32 pounds in 99 days, in another case to 33 pounds in 110 days; of the remaining 17 cases the loss of weight was small, and in many of them the wasting, which had previously been rapid, was more or less completely arrested. It may fairly be expected that if the drug can be brought into contact with the diseased tissue in a more concentrated form than when diffused throughout the blood, more decided results may yet be gained than have hitherto been possible from the administration by mouth. It has been estimated by Sternberg that the quantity of iodine required to prevent the development of a septic micrococcus in the blood of an adult man would be thirty-five grains. Now I have in one case administered thirty grains of iodoform daily, and no toxic symptoms were witnessed till after one month's continuous administration. It follows that, inasmuch as iodoform contains 96 per cent. of nascent iodine, the amount given approaches that which Sternberg's estimate shows to be necessary for complete disinfection of the blood. Treatment by injection into the focus of the disease in the lung appears to give us a means by which local developments may be reached by local treatment; and so complete local disinfection may be obtained, and the risk from the toxic action of the drug on the nerve centres may be avoided. It remains to be seen whether actual experience of the method will justify the expectations which theoretical considerations afford.

The first case is one of gangrene of the lung:

Simon T., 44, single, labourer in market garden. Had been out of work for six weeks, and suffering from exposure and want of food; in the habit of taking beer freely. Previous good health, with the exception of a dry cough for several winters. Was admitted to the Bristol Royal Infirmary on January 21st, 1885, complaining of shortness of breath and cough, but had been walking about till the day of admission.

*Pres. Cond.*—A well-nourished, prematurely aged man, cyanosed, breathing rapidly, with frequent short hacking cough. Expectoration frothy, blood-stained, and very fœtid. Chest barrel-shaped, with little movement, and very resonant percussion note; crepitation at both bases. Heart sounds weak; no murmur. Urine normal. No œdema of legs.

Ordered mist. cinchonæ, ammoniæ, c. ethere, quartis horis.

On 23rd, fœtor of breath very decided. Ordered creosote inhalations, and iodoform gr. ij, in pill, every four hours.

30th.—Patient very prostrate; marked hectic; expectoration profuse; fœtor constant, intensified on coughing; marked dulness on percussion below angle of left scapula; bronchial breathing, bronchophony, and coarse crepitation. Solution of iodoform in oil injected into the substance of the lung in the centre of the dull area at the left base; fifteen minims of olive oil, containing about one grain of iodoform, were injected with the hypodermic syringe. The injection appeared to have no immediate effect on the patient; it did not give rise to coughing, and there was no appearance of pain or other discomfort.

Feb. 1st.—Expectoration amounts to over a pint daily, and fœtor continues. Dulness at left base increasing; amphoric breath sounds and bronchophony, with much coarse crepitation. Injection repeated daily; it gave rise to no coughing or pain, and there was no hæmoptysis. On one occasion patient stated that he noticed the taste of the medicine in his mouth some hours after the injection. Was ordered one grain of quinine every four hours, and to continue the iodoform pill; brandy, 6 ounces.

Feb. 2nd.—Pulse very weak and small; great prostration. Some dulness and crepitation at the right base.

3rd.—Pulse better; patient less cyanosed. No dulness at the right base, but abundant coarse crepitation. Loud bronchial breathing at left base more extensive. Sputum examined for bacilli and for lung tissue; putrefactive bacteria abundant, but no tuberculous bacilli and no elastic tissue found. Solution of iodoform in ether was injected into the left lower lobe, ten minims containing two grains. Slight cough ensued immediately.

Feb. 6th.—Ethereal injections had been continued daily; patient made no complaint respecting them. He appears much improved, thinks he is better, sits up in bed, feeds himself, and looks brighter. The fœtor of breath is much diminished; now only perceived on deep coughing; does not pervade the air around. Cough is less harassing, and expectoration less in quantity. At left base bronchial breathing from angle of scapula downwards, and from spine to posterior axillary line. Percussion note close to spine high pitched, amphoric. Coarse gurgling on coughing. Bronchophony. At right base coarse crepitation, but no dulness. Vocal fremitus at left base diminished.

10th.—Injections continued daily. Patient improving in strength, sleeps better, has less cough, and expectorates about half a pint of frothy yellow muco-pus, slightly fœtid. Still has characteristic fœtor on deep coughing. Urine contains a trace of iodine.

13th.—Some complaint of giddiness, and therefore the iodoform pill was discontinued.

15th.—Patient looks brighter, is more active mentally, and seems stronger; takes food fairly. P., 124. Sputum a little fœtid; a few streaks of blood after the injection.

16th.—Seems weaker, more flushed. Ordered quinine, two grains, every four hours, and the iodoform injection was discontinued.

17th.—Slight delirium in the night. The fœtor a little increased on coughing.

18th.—Fœtor much increased.

19th.—More dyspnœa. Patient is very prostrate; face blue. Pulse, 130; resp., 46. Death took place in the evening.

*Post-mortem.*—Marked emphysema of both lungs. Left lower lobe firmly adherent and consolidated; tissue brittle, tearing on removal, and disclosing a cavity in posterior part near to the spine; the lung axis about three inches, and about two inches transversely, traversed by ribs of firm tissue, lined by a pseudo-mucous membrane; lung tissue around the cavity in state of grey hepatisation and friable; upper part of left lower lobe soft and œdematous, pitting on pressure; no fluid or slough in cavity, and no trace of the injected iodoform either in or around it. Right lower lobe congested and œdematous.

*Spleen.*—Numerous minute emboli dotted over the surface, looking like tubercles, but found to be triangular, with base on the surface.

Heart, kidneys, liver, normal.

In this case injections were given seventeen times at various parts of the lower lobe of the left lung into the consolidated and disintegrating area: four of these were solutions in oil, the remaining thirteen in ether. The effect was apparently beneficial, inasmuch as the temperature fell to normal, the expectoration diminished in quantity to about one-half and became much less offensive to the patient and his neighbours, and there was less cough.

The injections were discontinued in consequence of increasing weakness, which it was thought might be toxic. On the second day afterwards the fœtor of the breath had much increased, and so had the dyspnœa and prostration.

The diminution of the fœtor may fairly be attributed to the injections. The patient was at first a great annoyance to his neighbours, and became so again after the discontinuance of the injections.

The next case was one of chronic pneumonia:

William S. D., æt. 40, of good family history, but of intemperate habits, came under observation in December, 1884, and gave an account of failing health since the previous January. There had been two attacks of blood spitting, and profuse expectoration amounting to half a pint or more of pure pus daily, which did not contain tubercle bacilli or lung tissue. There was flattening at the right apex and contraction at the right base; the left supraspinous fossa was dull on percussion, and there was coarse bubbling crepitation at both bases. Liver dulness was normal. There was no albumen. Pulse, 96; small and weak. On January 23rd, 1885, a large patch of dulness and cavernous sounds were found at the base of the right lung: there was also coarse crepitation over the left lower lobe: the sputum amounted to one pint daily of pure pus, with no tubercle bacilli. The cavity in the right lower lobe was aspirated by Mr. Greig Smith; and on January 25th a drainage tube was introduced, but without giving exit to more than a few drops of pus, although air passed in and out freely. The tube was removed after four days, and the wound closed quickly.

Feb. 4th.—An ethereal solution of iodoform (gr. ij. in ℞ x.) was injected into the cavity through a hypodermic needle. The injection immediately set up violent coughing, and the patient tasted the ether, but the sputum did not become blood-tinged.

Feb. 19th.—Injections had been repeated five times in fifteen days. The taste of the ether had been again mentioned, but little cough was usually excited. On two occasions there had been momentary faintness immediately after injection. The sputum had diminished to half a pint, and no toxic symptoms were observed. The patient felt better, and went away to the seaside; the injections were therefore discontinued. They did not, however, effect any permanent improvement. The patient was of opinion that they diminished the expectoration, but this was only for a time: the process of disintegration of lung steadily progressed, and ultimately there were the usual signs of cavities on both sides, from the spine of the scapula on the right side and the angle of scapula on the left side downwards to the bases of the lungs. There was, however, no evidence of disease at either apex: at both infra-clavicular regions the breathing was puerile, and at no time were there moist sounds over the upper lobes in front. The sputum was examined on numerous occasions, but neither tubercle bacilli nor shreds of lung tissue could be found. There was usually a little fœtor, but not that of gangrene.

This patient died in June, whilst away from home, and I did not learn the details of his later history, but regret that the doubt as to the nature of his disease was not cleared up by *post-mortem* investigation.

The third case was one of chronic tubercular pleurisy. Nine injections were given into the dull area at the base of the right lung on nine successive days. The injections gave rise to no pain or cough, and there was no hæmoptysis. There was steady gain in weight whilst under treatment, the temperature became normal, cough and expectoration ceased, the dulness of right lung had much diminished, and the patient considered herself to be quite well.

Nellie S., 22, single, dressmaker. Admitted to Ward II., January 20th, 1885, for vomiting after food. Healthy till three months ago, when she was laid up with right pleurisy: has had cough ever since, and has been losing flesh steadily. Three days before admission vomiting commenced, and had continued daily. Catamenia had been regular. There had been no hæmoptysis. No history of consumption in the family; father and mother both living.

Dulness, crepitation, feeble breath sounds at right base from fifth dorsal vertebra downwards; breathing of bronchial character in axillary and scapular regions; diminished vocal fremitus below angle of scapula; dulness at right supraspinous and right infra-clavicular regions.

Heart sounds good. Temp. 101°. Urine, sp. gr. 1030; no albumen.

Jan. 28th.—Sickness subsiding. Patient can take milk freely. Patient has a cough, and expectorates a little muco-pus, which contains numerous tubercle bacilli. Temperature varies from 101° to 99°. Weight, 8st. 2lb. To take iodoform and quinine, of each one grain, every four hours. Right base continues dull, with faint breath sounds and diminished fremitus.

Feb. 4th.—Sickness has ceased. Patient is gaining weight, 12 pounds in seven days; now 9 stone. Temp. varies from 98·4° to 100°.

Feb. 6th.—Dulness at right base as before; crepitation at right supraspinous fossa; breath sounds weak over whole of right lung. To take two grains of iodoform, in pill, every four hours. Hypodermic needle disclosed the presence of clear serous fluid in the right pleura: the aspirator needle was introduced at the posterior axillary line in eighth intercostal space, but only a few drops of bloody fluid escaped.

Feb. 11th.—Weight, 9st. 2lb.

" 13th.—To have iodoform, gr. iij., every four hours.

" 17th.—Dulness at right base less decided. Little cough and expectoration. Patient not complaining of sickness or diarrhœa; less sweating at night. Temp. still rises at night to 100° or 101°. Iodoform, gr. j., dissolved in five minims of ether, was injected with hypodermic syringe below the angle of right scapula into the dull area. Patient made no complaint of the injection: it did not give rise to cough, but patient said she could smell something afterwards.

18th.—Ten minims of ether containing two grains of iodoform were injected, and this was repeated daily.

The injection gave no pain or cough: no complaint was made concerning it, and there was no hæmoptysis. Weight, 9st. 2lb.

March 5th.—Weight, 9st. 4lb. Patient is much better. Temperature is normal; no evening rise, no sweating. There is no cough or expectoration. Breath is rather short. To discontinue the injections and the pills, and to take cod-liver oil containing one grain of iodoform three times daily.

March 10th.—Weight, 9st. 6lb. Steady improvement. Made out-patient.

31st.—Weight, 10 stone. A little cough; no spitting whatever.

The fourth case was one of advanced tubercular phthisis of twelve months' duration, with much anæmia, œdema of legs, and albuminuria, with sub-normal temperature. Injections were made into the left upper lobe, at the infra-clavicular and superior axillary regions, on six occasions in nine days. There was no cough or hæmoptysis, but some complaint of pain round the left chest was made after the later injections. There was no change in the physical signs, and the weight remained stationary. She did not think it necessary to remain longer under treatment.

Ann G., 25, single, works at cotton factory. Father and mother died of consumption at about 50. Two brothers and one sister are healthy. Was quite well till a year ago; has since then been getting thin, has had bad cough for some months, and has been short of breath, but has never spat blood. Two months ago first noticed swelling of feet, and this has continued.

March 20th.—Anæmic. Weight, 7st. 1lb. Hæmic murmurs at base and in neck. Catamenia regular. Dulness and crepitation at left apex, front and back. Liver dulness in excess. Splenic dulness down to edge of rib. Some œdema of feet. Urine, 80 oz., 1009; neutral, pale, with one-fourth albumen. Ordered iodoform, gr. j., in pill, every four hours.

March 24th.—Œdema of legs subsided; little cough, no sputum. Temperature varying from 96·6 to 99·6.

March 31st.—Patient feels better. Weight, 7 stone. Taking iodoform, gr. ij., every four hours.

April 2nd.—No œdema. Temperature sub-normal. A little sputum was obtained, and was found to contain tubercle bacilli. Two grains of iodoform, dissolved in ten minims of ether, injected into the apex of left lung below the clavicle: it did not give rise to cough, nor did the patient taste the ether.

April 3rd, 5th, 7th, 9th, 10th.—Injections of iodoform repeated. The patient complained of pain round the left chest; accordingly the injection was discontinued. It did not give rise to cough or hæmoptysis.

April 12th.—Patient remains stationary in weight—7 stone.

15th.—Ordered iodoform, dissolved in cod-liver oil with creosote.

25th.—Gained one pound in weight.

28th.—Is much improved. Physical signs unchanged. Dull at left supraspinous fossa, and down to angle of scapula; in front, dull down to third rib. Dulness also below right clavicle and above it. No œdema. Not much cough; no expectoration. Urine varying from 1008 to 1010; albumen as before. Temperature, sub-normal. Made out-patient.

The fifth case was one of chronic tubercular phthisis, which had been under treatment for eighteen months as an out-patient, and had taken iodoform in pill or dissolved in cod-liver oil during nearly the whole of this period. The weight had increased from 8st. 2lb. in December, 1883, to 8st. 9lb. in September, 1884, after which time it remained stationary till May, 1885, when he began to lose weight, partly in consequence of sore throat, which gave rise to some dysphonia, although it did not result in laryngeal ulceration. Five injections were made from July 12th to the 22nd. The fluid was injected into the consolidated upper lobe of the left lung, at the supra scapular and infra-clavicular and superior axillary regions. The first injection of ten minims of the ethereal solution gave rise to cough, with momentary faintness and pallor. The following injections were limited to five minims, and no faintness ensued. Some pain and a localised pleuritic friction followed the third. The fifth was followed by neuralgic pain in the shoulder and up the neck. The temperature continued to be sub-normal, and the weight stationary.

Abraham L., æt. 31, married, shop assistant. Admitted July 11th, 1885.

Patient, previous to admission, had been under treatment as an out-patient for eighteen months. Treatment began on the 27th December, 1883, and has been continued up to the present date. When patient first came under notice he had been suffering from phthisical symptoms for eighteen months. During that time had a constant cough, with yellow nummular but scanty expectoration, which, on examination, was found to contain numerous bacilli. He complained also of general weakness. Never had any hæmoptysis or diarrhœa, and night sweats were only slight. Had not noticed any marked wasting, but said that he was not so stout as he was formerly. Voice husky at times. Patient's father died of phthisis.

Complexion of patient pale, but with no marked hectic flush. Fairly well nourished, and muscular tone good. Tendency to clubbed nails, but condition not absolutely typical. Pulse good. Heart normal. Abdominal organs healthy. Urine: Sp. gr. 1022; alk., no alb. Chest: Left side, dulness in front in infra-clavicular region for first three intercostal spaces, with some dulness also in supraspinous fossa behind. Loud bronchial breathing over area of dulness, with, at times, fine crepitation, best heard after coughing. Expiratory sounds were prolonged, and there was bronchophony, but no pectoriloquy. Vocal resonance and fremitus increased on left side. No evidence of existence of any cavity. No perceptible flattening of chest wall. Temperature normal.

WEIGHTS.

| | st. | lb. |
|---|---|---|
| December 27th, 1883 ... ... | 8 | 2 |
| January 21st, 1884 ... ... ... | 8 | 6 |
| February 4th, " ... ... ... | 8 | 6 |
| " 11th, " ... ... ... | 8 | 6 |
| " 25th, " ... ... ... | 8 | 6 |
| March 3rd, " ... ... ... | 8 | 7½ |
| " 24th, " ... ... ... | 8 | 8 |
| June 2nd, " ... ... ... | 8 | 7 |
| July 16th, " ... ... ... | 8 | 7 |
| August 11th, " ... ... ... | 8 | 9 |
| September 8th, " ... ... ... | 8 | 9 |
| February 16th, 1885 ... ... ... | 8 | 7 |
| March 30th, " ... ... ... | 8 | 8 |
| May 11th, " ... ... ... | 8 | 8 |
| June 22nd, " ... ... ... | 8 | 7 |
| July 16th, " ... ... ... | 8 | 5 |

*Treatment.*—Whilst an out-patient, from December 27th, 1883, to July 12th, 1885, patient was treated mainly with the pil. iodoform.; maximum dose, gr. ii. t.d.s. and cod-liver oil, sometimes alone and sometimes in combination with iodoform or creosote. Also had the Syr. Ferri. Hypophosph. Co. and tonics, with syrup to allay cough.

On December 1st, 1884, he had some symptoms of the toxic effects of iodoform, and iodine was found in considerable quantities in urine. Iodoform was discontinued for about six weeks.

July 12th.—On day after admission ♏ x. of the ethereal solution, containing gr. ii. of iodoform, were injected into the lung. The point of injection was just below the anterior fold of axilla, in the fifth intercostal space. During the injection patient began to cough, and soon felt faint and turned pale. No coughing after injection, and no spitting. Felt a little pain down the left side and left arm afterwards, and said that he could taste the iodoform.

13th.—Passed a good night. Very little cough and no spitting. Injection repeated, ♏ v. (gr. i.) in supra-spinous fossa. No cough or spitting, but felt a little faint. Patient did not on this occasion taste the injection; and it is doubtful whether it reached the lung, owing to the thickness of muscle.

14th.—Injection repeated below clavicle, second space, ♏ v. (gr. i.).

15th.—Injection, ♏ v., in axilla.

16th.—Complained of a good deal of pain in left side at seat of injection, and on auscultation House Physician detected a pleuritic friction rub. Injection was, therefore, not repeated to-day.

18th.—Injection, ♏ v.

22nd. " "

23rd.—Good deal of pain in chest, causing sleeplessness. No temperature and no signs of pleurisy. Expectoration very scanty, and cough slight; both diminished since admission. Weight, 8st. 5lb.

This series of cases is too small to enable one to draw any general conclusions: the results have not been decided, excepting from the negative point of view. They support the experience of Pepper, Fraenkel, Robinson and others, that injections into the lung substance are not dangerous. They also show that in many cases we may expect to relieve symptoms; and further, they encourage the hope that the method will become a familiar, an efficient, and a useful addition to our methods of treatment directed to the cure of chronic tubercular and other diseases of the lungs.

---

